# Quantum driven proton diffusion in brucite-like minerals under high pressure

**DOI:** 10.1038/s41598-020-64813-8

**Published:** 2020-05-15

**Authors:** Sofiane Schaack, Philippe Depondt, Simon Huppert, Fabio Finocchi

**Affiliations:** Institut des NanoSciences de Paris (INSP), Sorbonne Université, CNRS-UMR 7588, 75005 Paris, France

**Keywords:** Structure of solids and liquids, Theory and computation

## Abstract

Transport of hydrogen in hydrous minerals under high pressure is a key step for the water cycle within the Earth interior. Brucite Mg(OH)_2_ is one of the simplest minerals containing hydroxyl groups and is believed to decompose under the geological condition of the deep Earth’s mantle. In the present study, we investigate the proton diffusion in brucite under high pressure, which results from a complex interplay between two processes: the O–H reorientations motion around the c axis and O–H covalent bond dissociations. First-principle path-integral molecular dynamics simulations reveal that the increasing pressure tends to lock the former motion, while, in contrast, it activates the latter which is mainly triggered by nuclear quantum effects. These two competing effects therefore give rise to a pressure sweet spot for proton diffusion within the mineral. In brucite Mg(OH)_2_, proton diffusion reaches a maximum for pressures close to 70GPa, while the structurally similar portlandite Ca(OH)_2_ never shows proton diffusion within the pressure range and time scale that we explored. We analyze the different behavior of brucite and portlandite, which might constitute two prototypes for other minerals with same structure.

## Introduction

**Figure 1:**
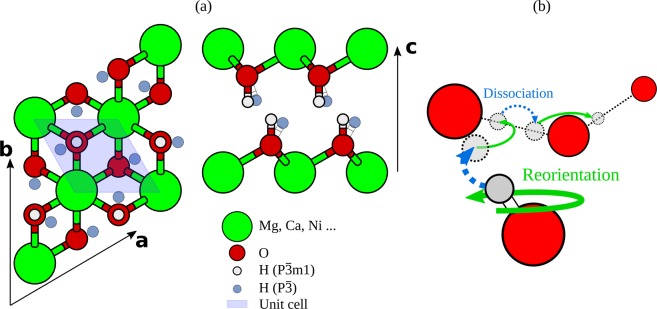
Left-hand side: description of brucite minerals structure. The primitive cell is shaded; the simulation box is defined in-plane by vectors ${\bf{a}}=a\mathrm{(3/2,}\sqrt{3}\mathrm{/2,0)}$ and ${\bf{b}}=a\mathrm{(0,}\sqrt{3}\mathrm{,0)}$, and contains 3 Mg(OH)_2_ units. The hydrogen atoms in the P$\bar{3}m1$ symmetry structure (light grey) are in the $2d$ Wyckoff sites, while the hydrogen atoms in the P$\bar{3}$ structure (dark grey) are in the disordered $6i$ Wyckoff sites. The latter is the stable structure at the pressures of our simulations. Right-hand side: Proton diffusion mechanism which requires a succession of two alternate steps: proton reorientation (green arrows) i.e. O–H rotation around $c$; and dissociation (blue arrows) i.e. proton hopping between oxygen layers. Oxygen atoms are colored in red, hydrogen atoms in grey. The size of atoms decreases with depth.

Hydroxide minerals play an important role in several problems in geology, surface science or for industrial applications. Among them, brucite Mg(OH)_2_ can be formed at the interface between periclase MgO and water at ambient conditions^1–3^. The trigonal brucite structure consists of alternating layers along the ${\bf{c}}$ axis that terminate with hydroxyls (Fig. 1). This structure is common to other hydroxides of divalent metals, such as Ca(OH)_2_, Ni(OH)_2_ and Cd(OH)_2_. Portlandite Ca(OH)_2_ is the main component of cements and concretes, which motivated a large number of investigations about its elastic properties. Because of their anisotropic structure, brucite isostructural minerals are much more compressible along the ${\bf{c}}$ axis than in the other two directions, parallel to the stacks.

Mg(OH)_2_ can also act as a water vector in subduction zones, through complex processes that take place within the Earth interior^4,5^. Therefore, the behavior of brucite and brucite-like minerals at very high pressure has been widely investigated. X-ray diffraction of Mg(OH)_2_ up to 78 GPa showed that the $c/a$ ratio decreases steadily from ambient pressure up to about 25 GPa and then stays almost constant^6^. Those results suggest that the properties of brucite at very high pressure, and in particular the nature of the inter-layer bonding, could differ significantly from ambient conditions. Moreover, the hydroxyl groups that are parallel to the ${\bf{c}}$ axis at ambient conditions slant in three equivalent positions as the inter-layer distance shrinks even under moderate pressure^7^ or when decreasing temperature^8^. Besides reducing the global symmetry from P$\bar{3}m1$ down to P$\bar{3}$, the slanted OH groups can significantly alter several physical properties of brucites. Firstly, it could allow for the formation of hydrogen bonds between the layers^9^, which eventually reinforce under further compression and modify the compressibility along $c$^10^. Secondly, when the protons form a non null $\theta$ angle with the ${\bf{c}}$ axis, they cannot arrange in a static ordered structure. Such proton disorder, which is closely related to *proton frustration*^11,12^, has also been invoked as the reason for the pressure-induced hydrogen sublattice amorphization in brucites^7,13^. The existence of a quasi two-dimensional proton liquid in those extreme conditions can be conjectured, but the properties of the whole structure, if stable, have so far escaped a precise characterization. In particular, the occurrence of proton hopping is plausible, but whether this process results in a long-range diffusion is a totally open question.

From the theoretical viewpoint, the previous observations call for a dynamical treatment of the proton arrangement within the brucite structure at high pressure. Moreover, in such conditions nuclear quantum effects, that is, all the properties that go beyond a purely classical description of ion dynamics^14^, such as zero-point energy, ion delocalization and tunneling, can have an important impact on the brucite properties. Its light mass makes hydrogen prone to fast diffusion and subject to significant quantum effects even at room temperature. In particular, the high O–H stretching frequency implies a non-negligible zero-point energy of about 0.2 eV that is crucial when hopping through local sites which are separated by barriers that classical nuclei cannot overcome simply by thermal fluctuations^15–17^.

Despite the complexity and computational cost of accounting for the quantum nature of light nuclei in simulations, efficient methods have arisen^18–21^, and, stimulated by the increasing interest in often paradoxical nuclear quantum effects (NQE)^14^, a new field is rapidly growing with potential applications in an eclectic collection of issues including protonic conduction^22^, hydrogen in biological matter^23^, water circulation in Earth’s mantle^24,25^ or hydrogen storage^26,27^.

In this work, we address the proton diffusion process occurring in brucite Mg(OH)_2_ that appears to be a specific case as compared with portlandite Ca(OH)_2_, which was the object of a recent theoretical study^28^, and is also discussed here. The reasons for the different behaviors of these two compounds are elucidated. We employ classical and Path-Integral Molecular Dynamics^29–31^, (PIMD) to disentangle thermal from nuclear quantum effects in a consistent framework. Among the various proton diffusion mechanisms that were considered in the past, we confirm that the *relay mechanism*^28,32^, is at play in brucite under high pressure. This two-step process involves proton hops from one oxygen atom to another and rotations of OH groups. We also show in the following that proton hopping is quantum driven while OH rotation is mostly thermally activated.

## Structural Changes Under Pressure

The brucite minerals are layered structures composed by stacks of metallic ions, oxygen, and hydrogen atoms in a CaI2-type structure^11,12,28^. The metallic element (Mg, Ca, Ni …) has an impact on several physical parameters, in particular, the lattice parameters and the compressibility of the system are different in the two systems we studied: brucite (Mg(OH)_2_) and portlandite (Ca(OH)_2_)^10^. The metal layers terminate with hydroxyl groups on both sides (Fig. 1, left). At ambient pressure and temperature, the brucite minerals belong to the P$\bar{3}m1$ space group with hydrogen atoms located on the threefold axis above or below oxygen atoms ($2d$ Wyckoff sites) with full occupancy as shown in the left-hand side of Fig. 1.

However, in accordance with previous experimental and simulation results^7,11,12^, as pressure increases, due to the growing repulsive interaction between protons on opposite layers, the protons do not remain above the corresponding oxygen atom. Brucite minerals adopt a P$\bar{3}$ configuration, where the H nuclei are in the $6i$ Wyckoff sites with a 1/3 occupancy factor (Fig. 1, left). This proton rearrangement has two noticeable consequences: first, a frustration of the proton orientation upon compression^11,33,34^ second, the protons in $6i$ sites could form hydrogen bonds between distinct layers^7^. This point, which has been raised by several authors, is discussed in the following.

The stability of both brucite and portlandite upon compression has been investigated in the past and an amorphization of portlandite was found through infra-red absorption to occur between 10 and 15 GPa^33^. In brucite, however, X-ray experiments^6^ up to 78 GPa at 600 K showed a smooth variation of the diffraction patterns; in the same paper, the authors deduced an equation of state according to which brucite would decompose into periclase and water around 27 GPa at ambient temperature^6^. These apparently contradictory results suggest a significant kinetic decomposition barrier. More recently, a phase transition toward a tetragonal structure was suggested in brucite above 20 GPa by ab initio simulations^35^; nonetheless, up to now, no such transition was observed experimentally to our knowledge^6,34^. The pressure increase shrinks the distance between the MgO layers, due to the important compressibility^10^ along the ${\bf{c}}$ axis, thus allowing the formation of inter-layer hydrogen bonds. This effect is expected to enhance the cohesion of the system and thus to stabilize the structure, which might be connected to the existence of a rather large barrier hindering brucite to decompose. Moreover, the formation of inter-layer hydrogen bonds could allow the protons to hop between the two oxygen atoms on the facing Mg(OH)_2_ layers.

### Proton diffusion mechanism

As in other hydrous minerals^24,25^, hydrates^36^ and ices^15,37^, the proton can hop along hydrogen bonds. Following Dupuis *et al*.^28^, we refer to this process here as dissociation, as it implies the breaking of a covalent O–H bond to form another distinct O–H covalent bond. However, another process, namely the reorientation (the hopping between the three $6i$ Wyckoff sites in the P $\bar{3}$ space group), is necessary for the proton to move away from the initial O site through the crystal. Only the combination of dissociation and reorientation can drive proton diffusion; one of the two processes alone would simply imply a back-and-forth proton motion between neighboring sites. Proton diffusion in brucite-like minerals is therefore a two-step compound process, intrinsically different from the Grotthuss mechanism that is active in water and other H-bonded hydrates^32,38^.

In P$\bar{3}$ Mg(OH)_2_, the proton experiences an effective triple well potential among the $6i$ Wyckoff sites, which controls reorientation within the ($ab$) plane. We refer to these reorientation events as “in-plane” motion. On the other hand, the “out-of-plane” dissociation mechanism involves an effective double well potential along the O-O direction characterizing the covalent and hydrogen bonds. A recent study^28^ analyzed the proton diffusion mechanism in portlandite for different temperatures and concluded that thermal activation of the reorientation hopping can be the limiting factor for proton diffusion. Although the authors guessed that nuclear quantum effects could favor the dissociation mechanism, the latter process was studied only classically.

## Results

In the following, we mainly focus on the effect of pressure in brucite and unravel the complex and quantum driven proton diffusion in this material, accounting for NQE in a consistent framework for both reorientation and dissociation processes. Rising pressure tends to increase the strength of hydrogen bonds. As a consequence, OH dissociation and proton reorientation display opposite trends with pressure. Furthermore, we show that dissociation is significantly enhanced by NQE while reorientation is mostly thermally activated. Finally, the behavior of protons in portlandite is analyzed, and differences between Mg(OH)_2_ and Ca(OH)_2_ are discussed.

### Quasi-2D proton layer

**Figure 2:**
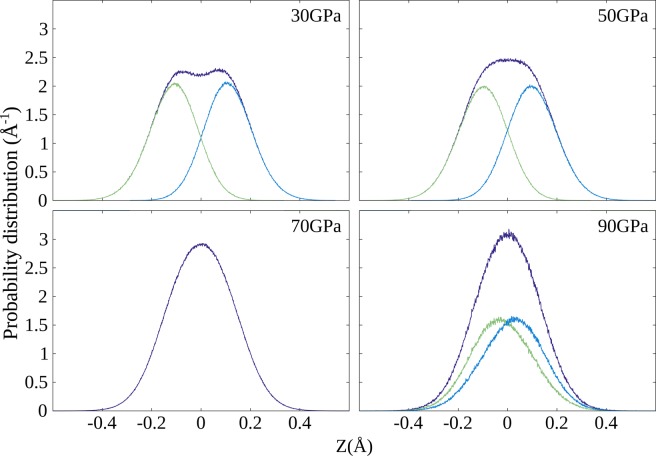
Probability distribution of the proton position along ${\bf{c}}$. The green lines relate to the bottom layer of hydrogen nuclei (Fig. 1), the blue lines to the top layer, while the purple lines are the sum of both.

Before addressing proton diffusion, we need to examine the structural changes of the hydrogen layers under increasing pressure and the possible formation of an almost two-dimensional proton layer. Figure 2 shows the probability distribution of protons along the ${\bf{c}}$ direction. Initially, each proton lies either in the upper or lower plane – we therefore label the protons according to their initial location and compute the corresponding “lower-layer” or the “upper-layer” proton distributions.

As pressure increases, the overall distribution width decreases, the double-peak structure still visible at 30 GPa disappears as the lower- and the upper-layer distributions tend to merge (see Fig. 2). Accounting for NQE via PIMD is crucial for the study of this process: zero-point energy yields an important contribution to the proton distribution width and makes the merging of the two layers occur at lower pressure than for classical protons.

An other interesting effect appears when considering hydrogen motion between the two layers. At the lowest pressures (30 and 50 GPa), the bottom and top layer protons do not mix: although proton hopping (either of a few beads or of the whole ring-polymer in the PIMD framework) from one layer to the other does occur, the protons always return to their original layer by a second hop in reverse. Protons initially situated on one layer thus remain on that layer for the whole duration of the simulation with, from time to time, a short exploration of the other layer. However, at 70 GPa, lower and upper layers are not distinguishable as reverse hopping does not always follow: protons do not belong to one particular layer anymore but rather all protons should be considered as forming a single quasi-2D plane within which efficient diffusion can occur, as we further analyze in the following. Interestingly at the highest probed pressure, 90 GPa, the overall distribution becomes narrower, but the lower and upper protons can again be distinguished: as for the lowest two pressures, they tend to stay in their initial layer with only occasional hops, despite a large overlap between the partial distributions. This behavior will be rationalized in the following, examining the different pressure trends of the two hopping mechanisms that are necessary for long-range proton diffusion.

### In-plane reorientation

**Figure 3:**
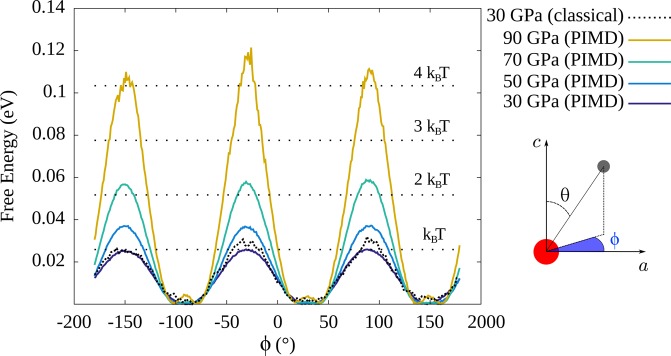
Free energy profile along the polar angle $\phi$. The proximity between classical and PIMD profiles at 30 GPa shows that the reorientation mechanism is dominated by thermal effects. The thermal energies corresponding to $T=300$ K and higher temperatures are also shown as long-dotted lines for comparison.

First we discuss the reorientation mechanism. As already described, within the P$\bar{3}$ structure, protons hop in-plane between the $6i$ sites. This motion can be efficiently described by the azimuthal angle $\phi$ as shown in the sketch of Fig. 3. From the probability distribution of the latter ${{\mathscr{P}}}_{{\rm{r}}}(\phi )$, we extracted the Gibbs free-energy profile $G=-\,{k}_{B}T\,\log \,{{\mathscr{P}}}_{{\rm{r}}}(\phi )$, which includes both thermal and quantum effects.

As shown in Fig. 3, the proton free-energy profile along this coordinate has a three-fold symmetry with equivalent barrier heights between the three wells, consistently with the P$\bar{3}$ configuration. The barrier width of this free energy profile is grossly proportional to $\frac{2\pi }{3}{d}_{{\rm{O}}-{\rm{H}}}\,\cos \,\theta$ ($\theta$ being the zenith polar angle and ${d}_{{\rm{O}}-{\rm{H}}}$ the covalent O–H bond length), that is, how far the O–H bond slants away from the ${\bf{c}}$ axis.

Upon compression, we observe that the free energy barrier height increases, from 20 meV at 30 GPa up to 100 meV at 90 GPa, revealing a pressure induced confinement of the proton along this coordinate. This is in line with the increasing of the average polar angle $\theta$ with pressure that splits the $6i$ sites apart. Along with the compression of the layers along the ${\bf{c}}$ axis, the protons from the two layers try to minimize their mutual repulsion by increasing $\theta$. As a consequence, the reorientational dynamical disorder, thermally activated at low pressure, tends to slow down.We point out that classical simulations, not including NQE, yield almost identical ${{\mathscr{P}}}_{{\rm{r}}}(\phi )$ distributions, meaning that the quantum behavior is in this case moderate within the pressure range that we explored. The effect of pressure contrasts with that of temperature, which tends to allow the proton to explore equivalently the three wells^28^ by thermal activation.

### Out-of-plane dissociation

As the hydrogen planes become closer upon compression, the protons in $6i$ sites adopt quasi-linear O–H–O configurations, where the O anions belong to distinct Mg(OH)_2_ stacks. This configuration is thus prone to the formation of inter-layer hydrogen bonds. It has been shown^12^ that only weak hydrogen bonds could be present in brucite. However, as we discuss below, a double-well potential is found along the O-O direction at moderate pressure, which suggests the onset of hydrogen bonds and occurs in parallel with the pressure-induced creation of quasi-2D proton layers in the structure. All along those transformations, NQE play an important role.

**Figure 4:**
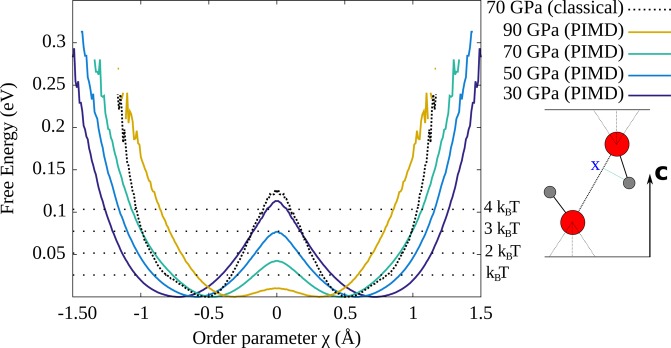
Proton free energy profile along the order parameter $\chi$ as defined in the text and sketched on the right (O in red, proton in gray).

In order to investigate the proton effective potential, we adopt as the order parameter^17^ $\chi$ which is the difference between the distances that separate the hydrogen atoms from their nearest and second nearest neighbor oxygen atoms projected on the O-O direction (see sketch in Fig. 4).

1 $$\chi =|\overrightarrow{{{\rm{O}}}_{2}{\rm{H}}}\cdot {\overrightarrow{u}}_{OO}|-|\overrightarrow{{{\rm{O}}}_{1}{\rm{H}}}\cdot {\overrightarrow{u}}_{OO}|$$

with ${\overrightarrow{u}}_{OO}$ the unitary vector in the O_1_–O_2_ direction.

The free-energy profiles along this coordinate (see Fig. 4) show that: (i) the effective potential is a double well with minima at $\pm {\chi }_{0}$, as for other hydrogen-bond forming crystals^17,24^; (ii) the barrier height decreases upon compression, from 4 ${{\rm{k}}}_{{\rm{B}}}{\rm{T}}$ at $P=30$ GPa to 0.5 ${{\rm{k}}}_{{\rm{B}}}{\rm{T}}$ at $P=90$ GPa. This occurs while the O-O distance shrinks with pressure along the ${\bf{c}}$ axis, bringing the two proton equilibrium positions closer and reducing $|{\chi }_{0}|$. At high pressures, a proton can therefore hop from one oxygen atom to another through quantum tunneling, zero-point energy or thermal activation, which constitutes the so-called “dissociation” process^28^. The difference between classical and quantum simulations is significant as the classical barrier is $\sim 3{{\rm{k}}}_{{\rm{B}}}{\rm{T}}$ higher than its quantum counterpart at 70 GPa. Therefore, while the proton reorientation mechanism is thermally activated, the dissociation process is mainly quantum driven.

### Proton diffusion sweet spot

**Figure 5:**
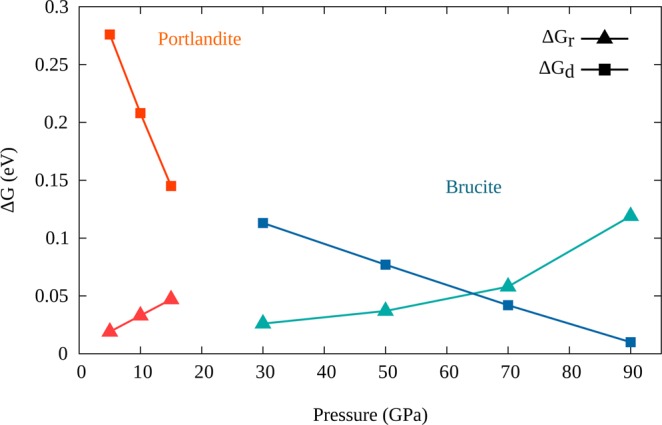
Free energy barriers for brucite and portlandite, as computed from the probability distributions for reorientation ($\Delta {G}_{r}$) and dissociation ($\Delta {G}_{d}$). As discussed in the text, the barriers for proton reorientation and diffusion show opposite trends with increasing pressure, with a non-linear increase of the reorientation barrier in Mg(OH)_2_. The competition between the two trends generates a pressure sweet spot in brucite between 60 and 70 GPa, where both reorientation and dissociation barriers can be overcome; in this pressure range, proton diffusion is enhanced, which requires both mechanisms to occur. Such a sweet spot cannot be reached in portlandite due to the amorphization transition taking place between 10 and 15 GPa.

The evolution of the free energy barrier heights upon compression is reported in Fig. 5. In the case of brucite Mg(OH)_2_, the barrier height for dissociation decreases from $\Delta {G}_{d} \sim 0.11$ eV at 30 GPa to $\Delta {G}_{d} \sim 0.01$ eV at 90 GPa. In contrast, the reorientation barrier increases from $\Delta {G}_{r} \sim 0.03$ eV to $\Delta {G}_{r} \sim 0.1$ eV within the same pressure range. The two curves cross at $\sim$70 GPa where hopping rates for the two processes have the same order of magnitude, while reorientation dominates at lower pressures and dissociation prevails at higher pressures. We thus suggest that the highest proton mobility occurs around $P=70$ GPa, which represents the sweet spot for proton diffusion.

We provide in Table 1 a rough estimate of both dissociation and reorientation reaction rates ${\kappa }_{d}$ and ${\kappa }_{r}$, through the Eyring-Polanyi equation^39,40^:

2 $${\kappa }_{d,r}=\frac{{k}_{B}T}{h}{{\rm{e}}}^{-\frac{\Delta {G}_{d,r}}{{k}_{B}T}}$$

**Table 1:** Eyring-Polanyi inverse reaction rate for the dissociation ${\kappa }_{d}^{-1}$ and reorientation ${\kappa }_{r}^{-1}$ mechanisms, computed for brucite (left) and portlandite (right).

		Brucite Mg(OH)_2_				Portlandite Ca(OH)_2_			
$P$	(GPa)	30	50	70	90	5	10	Transition	15
${\kappa }_{r}^{-1}$	(ps)	0.43	0.65	1.47	12.3	0.32	0.54		0.95
${\kappa }_{d}^{-1}$	(ps)	13.2	3.07	0.83	0.24	8.63 $\times {10}^{3}$	438		44.3

The temperature is $T=300$ K. The transition pressure for amorphization of portlandite is reported from refs. ^13,42^.

While in brucite the typical reorientation and dissociation times are reachable within our simulation duration, in portlandite (discussed in greater detail below), because of the larger barriers with respect to brucite (see Fig. 5), proton dissociation characteristic time in Ca(OH)_2_ is on the ns scale or even larger for all pressures before the amorphization transition. We note in passing that ${\kappa }^{-1}$, as evaluated for classical protons within the Eyring-Polanyi equation, is several order of magnitudes greater.

### In-plane proton distribution

**Figure 6:**
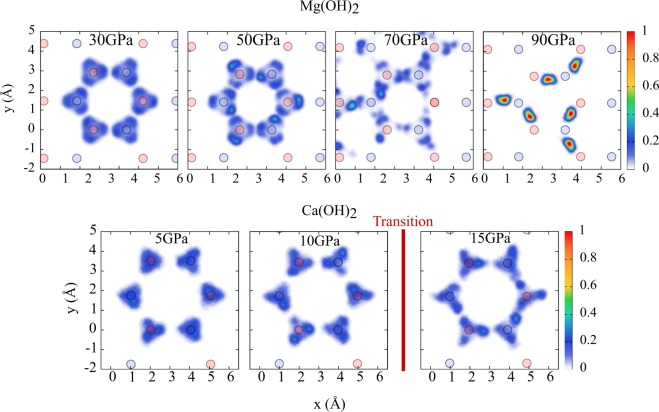
Distribution of the proton positions in the $(x,y)$ plane at 30, 50, 70 and 90 GPa for brucite Mg(OH)_2_ (upper panels) and portlandite Ca(OH)_2_ (lower panels). The circles represent the projection of the oxygen sites on the $(x,y)$ plane (light red: bottom layer, light blue: top layer). Periodic boundary conditions are not used in computing this distribution in order to visualize the displacement of the protons: in brucite, the protons that were initially located within the simulation box move out of its boundaries at 70 GPa, which is an indication of diffusion occurring even within the 30 ps duration of our simulations. In portlandite, this might happen at pressures above 15 GPa, thus beyond the amorphization transition pressure that is evidenced by the red vertical line.

The barrier height analysis above is perfectly consistent with the probability distribution of the proton positions in the $({\bf{a}},{\bf{b}})$ plane as shown in Fig. 6. For $P=30$ GPa, the proton distribution shows three broad peaks next to each oxygen atom, thus revealing reorientation processes between the $6i$ sites. As pressure is increased to 50 GPa, the peaks become narrower and centered at a greater distance from the oxygen sites. This is due to the hindering of the reorientations and the progressive slanting of the O–H bonds towards the $({\bf{a}},{\bf{b}})$ plane. In addition, the proton density midway between the oxygen sites increases as dissociation become easier. At $P=70$ GPa, we observe evidence of a long-range proton diffusion process as the hydrogen nuclei spread all over the simulation box. The onset of dissociation, while reorientation processes still occur, allows the protons to migrate beyond their first neighbors. The range of this in-plane motion (over several Å) can be compared with the width of the out-of-plane ${\bf{c}}$ axis distribution as in Fig. 2 (approximatively 0.2 Å), clearly indicating the two-dimensional character of this process. Finally, at the highest pressure, $P=90$ GPa, although the dissociation probability keeps increasing, the reorientation processes are locked in. The computed proton distribution is not symmetric among the $6i$ sites anymore and long-range proton diffusion is hindered. This confirms the specificity of the 60–70 GPa pressure range as a sweet spot for proton diffusion.

Due to the relative shortness of the PIMD runs and the fact that a Langevin thermostat with a friction coefficient of 10 ps^−1^ is attached to the centroid motion to achieve efficient sampling (see Methods section), the PIMD simulations cannot be used to obtain a direct estimate of the proton diffusion coefficient. Nonetheless, the trends displayed on Fig. 6, with the onset of efficient proton diffusion at the sweet spot pressure of 70 GPa are qualitatively correct and consistent with the previous discussion. Furthermore, the PILE-L thermostatting scheme^31^ that we use in this work reduces, in the limit of a vanishing centroid friction, to the Thermostatted Ring-Polymer Molecular Dynamics (TRPMD) algorithm^41^ for computing dynamical observables. We expect centroid thermostatting to hinder proton diffusion in our PIMD dynamics, which explains why long-range proton diffusion is not directly observed at 50 GPa, even though the estimated dissociation and reorientation inverse rates are relatively short compared to the PIMD trajectory length. Note that centroid thermostatting does not affect the PIMD estimates for static properties such as the free energy barrier $\Delta G$, and therefore it has no impact on the rates presented in Table 1. Proton diffusion should indeed occur already at pressures lower than 70 GPa and it might be at the root of a de-stabilization of the system easing the suggested^35^ phase transition, or could be involved in the water transfer in the earth mantle.

### Comparison with portlandite

Finally, we close our discussion on proton diffusion in brucite-like minerals by a comparison with portlandite Ca(OH)_2_ which has the brucite structure for pressures up to approximately 15 GPa. The same analysis as for brucite was done systematically for portlandite. In Fig. 5 we present the evolution of free energy barriers of the proton reorientation and dissociation mechanisms. We observe that in portlandite the reorientational barrier $\Delta {G}_{r}$ at 10 GPa is comparable to that in brucite at 50 GPa. However, the pressure effect on the latter barrier is more important in portlandite as shown by the larger pressure slope of 2.8 meV/GPa while its counterpart in brucite is about 1.6 meV/GPa. Ca^++^ cations are larger than Mg^++^ ones, so that the computed in-plane O-O distance at $P=10$ GPa is $\simeq$ 3.45 Å in portlandite and $\simeq$ 3.06 Å in brucite; in contrast, the distance between the oxygen anions on distinct stacks is almost the same in the two crystals. This implies larger polar angles $\theta$ in portlandite than in brucite, which efficiently hinder the reorientation mechanism, even at relatively low pressures.

As far as the dissociation barriers $\Delta {G}_{d}$ are concerned, in portlandite they are greater than in brucite but decrease much faster with pressure. The estimated pressure slope $\frac{\partial \Delta {G}_{d}}{\partial P}$ is 14 meV/GPa in portlandite, as compared to 2 meV/GPa in brucite. This derives from the larger compressibility of portlandite with respect to brucite, as demonstrated in a recent work^10^. It has to be noticed that the large value of the dissociation barrier in portlandite would require very long runs of path integral ab initio molecular dynamics, much beyond the scope of this work, to record a significant number of events. Nevertheless, we detected a few events as some of the replicas of the PIMD simulations did occasionally reach the barrier top. A precise evaluation of the barrier heights would have nevertheless required the use of accelerated sampling techniques, such as in ref. ^42^.

Finally, the crossing point of the dissociation and orientational barriers in portlandite should occur beyond 20 GPa, with diffusion barriers comparable to that of brucite at 70 GPa. However, a transition towards an amorphous phase is reported between 10 and 15 GPa^13,43^, and our own simulations reveal instability of the system at 20 GPa. Therefore, as shown in Fig. 6, no diffusion was observed for portlandite within the time scale of our simulations. Indeed, the reaction rate estimates, given in Table 1, yield much longer times than in brucite.

The values reported in Table 1 for portlandite agree remarkably well with the results of Dupuis and coworkers^28^ as regards the reorientation rate at 10 GPa and its increase with decreasing pressure. Our results for the dissociation rate, however, are lower than that of ref. ^28^ by as much as 3 orders of magnitude under comparable thermodynamic conditions. In ref. ^28^, different methodologies were used for the computation of the two rates: whereas path integral methods were employed for the reorientation process, metadynamics with classical nuclei was used for dissociation. In this work on the other hand, we compute the two rates within a fully consistent framework: the same PIMD trajectories are employed to estimate the free energy barriers associated with the two processes, which are then inserted into the Eyring-Polanyi approximation. We do not resort to any type of accelerated sampling scheme to overcome the barriers. Note that for the lowest pressure of 5 GPa, the ring-polymer beads very rarely explore the top of the dissociation barrier, therefore the uncertainty on $\Delta {G}_{d}$ is large (we estimate $\Delta {G}_{d}=\pm$ 20 meV). This accuracy is nonetheless sufficient to exclude, within our approach, such a fast dissociation as reported in ref. ^28^.

It can be noted, that among the other systems sharing the same structure as brucite, our preliminary analysis of theophrastite Ni(OH)_2_ indicates that this system should also present a sweet spot for proton diffusion at approximately the same pressure as Mg(OH)_2_, due to comparable ionic radii between Mg^++^ and Ni^++^ cations.

## Conclusion

To summarize, we analyzed the proton diffusion mechanism in both brucite (Mg(OH)_2_) and portlandite (Ca(OH)_2_) under pressure, taking into account nuclear quantum effects by path-integral based ab initio molecular dynamics. Proton diffusion in those crystals involves two stages to occur: a reorientation motion within the (${\bf{a}}$,${\bf{b}}$) plane, and a proton dissociation between two oxygen atoms on opposite layers. Firstly, we have seen that the reorientation mechanism is thermally activated and that the pressure tends to localize the proton in a certain orientation, making the reorientation motion less likely. Secondly, in contrast with the reorientation, we showed that the dissociation mechanism was quantum driven and was made easier by increasing pressure through the formation of a quasi two-dimensional hydrogen layer.

These two antagonistic effects give rise to a pressure sweet spot for proton diffusion within 60 and 70 GPa in brucite. However, proton diffusion could also occur at much lower pressure, although it is less probable, and could be at the root of a destabilization of the structure, as suggested by the theoretical predictions of a phase transition^35^ at 20 GPa or decomposition into MgO and H_2_O at the same pressure^6^. Beyond this pressure threshold the reorientation becomes a bottleneck for proton diffusion, while dissociation is the rate-limiting step at lower pressure.

Finally, by systematic comparison with portlandite, we demonstrate the specificity of brucite for proton diffusion. Indeed, the proton diffusion sweet spot in portlandite would occur at pressures well beyond its transition toward an amorphous phase.

## Methods

Molecular dynamics (MD) simulations were carried out at room temperature and fixed volume either via a classical Langevin equation or within the Path Integral (PI) framework to take into account nuclear quantum effects while the electronic structure and atomic forces were described within the Density Functional Theory (DFT). We compute the electron density and atomic forces within the Perdew-Wang Generalized Gradient approximation to the exchange and correlation density functional^44^, using the Quantum Espresso package^45^ in combination with the i-PI^46^ interface for the path-integral simulations. We employed ultra-soft pseudo-potentials with a plane wave expansion cutoff of ${E}_{cut}=50$ Ry for the Kohn-Sham states and 8 times as large for the charge and the potential, ensuring total energy convergence. Both brucite Mg(OH)_2_ and portlandite Ca(OH)_2_ were simulated by using a hexagonal $(\sqrt{3}\times \sqrt{3}\times \mathrm{1)}$ supercell and a $\mathrm{(2}\times 2\times \mathrm{2)}$ k-point sampling centred at $\frac{2\pi }{a}\left(\frac{1}{2},\frac{1}{2},\frac{a}{2c}\right)$.

The number of beads in the PIMD simulations was set to 24 and checked to provide convergence of kinetic and potential energies. The PIMD simulations were performed using the efficient PILE-L thermostatting scheme^31^ with a centroid friction coefficient of 10 ps^−1^. Lattice parameters were obtained through systematic volume relaxation of the system ensuring isotropic stress tensors for each pressure. The optimized equilibrium lattice parameters $a$ and $c$ were remarkably similar between classical and path-integral MD simulations for the pressures here considered: the differences between the classical and quantum results for $a$ and $c$ amounted to few hundredths of Å. Finally, the typical duration time of the simulations was 30 ps.
